# Effects of pulmonary rehabilitation in people with lung cancer after lung resection: a systematic review and meta-analysis

**DOI:** 10.1007/s11764-025-01952-9

**Published:** 2026-01-30

**Authors:** Qun Wang, Zhe Liu, Lei Qi, Sek Ying Chair, Ruitong Gao

**Affiliations:** 1 Shenzhen University, Shenzhen, China; 2 City University of Hong Kong, Hong Kong, China; 3 Qilu Hospital of Shandong University, Jinan, China; 4 Chinese University of Hong Kong, Hong Kong, China

**Keywords:** Pulmonary rehabilitation, Lung cancer, Lung function, Exercise capacity, Quality of life, Meta-analysis

## Abstract

**Purpose:**

This systematic review and meta-analysis aims to evaluate the effectiveness of pulmonary rehabilitation in improving physical and psychological outcomes after lung resection.

**Methods:**

A literature search was conducted across five electronic databases, identifying randomized controlled trials evaluating pulmonary rehabilitation in lung cancer patients after lung resection. Screening, data extraction, and quality assessment were conducted independently by two reviewers. The revised Cochrane risk-of-bias tool for randomized trials was used to evaluate the quality of included studies. Data synthesis was performed using RevMan Web, with results visualized in forest plots.

**Results:**

Eighteen studies (*n* = 1795) were included; only three were rated as having low risk of bias. The meta-analysis revealed that pulmonary rehabilitation, including exercise and breathing training, significantly improved lung function, such as the forced vital capacity, physical capacity, 6-min walking distance, and the physical domain of quality of life in post-surgery lung cancer patients compared to controls. Subgroup analysis indicated that interventions lasting 12 weeks or longer were associated with greater improvements. Egger’s test suggested no significant publication bias.

**Conclusions:**

The findings demonstrate the positive effects of pulmonary rehabilitation, including exercise and breathing training, on postoperative recovery in lung cancer patients. Significant improvements were observed in lung function, exercise capacity, and quality of life.

**Implications for cancer survivors:**

Our systematic review underscores pulmonary rehabilitation as an effective intervention to improve physical function and quality of life in lung cancer patients following lung resection. It supports broader adoption to enhance postoperative recovery and long-term health in these patients.

**Trial registration:**

PROSPERO (reference: CRD42024616958).

**Supplementary Information:**

The online version contains supplementary material available at https://doi.org/10.1007/s11764-025-01952-9.

## Introduction

According to Global Cancer Statistics 2022, which reported data from 185 countries, lung cancer was the most frequently diagnosed cancer worldwide, accounting for around 2.5 million new cases (12.4% of total cases) [1]. Lung cancer was also the leading cause of cancer-related mortality globally, responsible for an estimated 1.8 million deaths (18.7% of total cancer deaths) [1]. In China specifically, the latest data from the National Cancer Center indicates that lung cancer was the most prevalent cancer in 2022, with 1,060,600 new cases. It was also the most common cancer among both men and women. For both sexes combined, lung cancer remained the primary cause of cancer-related death, with 733,300 deaths reported [2]. The huge number has imposed a significant health burden on the healthcare system and society.

Lung resection is a widely utilized treatment for lung cancer patients. Advances in early presurgical staging, surgical techniques, and adjuvant therapies have led to improved survival rates [3]. For patients with stages I and II lung cancer, the 5-year survival rate has increased to between 84.8% and 87.3% [4]. However, lung cancer surgery is related to functional limitations due to various factors during surgery and patient characteristics, which can severely impact postoperative quality of life (QoL) of the patients [5, 6].

Pulmonary rehabilitation has been widely used in treating chronic respiratory diseases and may be a valuable approach to improving the QoL and overall health of patients and survivors of lung cancer [7, 8]. According to the American Thoracic Society/European Respiratory Society, pulmonary rehabilitation is defined as a “comprehensive intervention based on a thorough patient assessment followed by patient-tailored therapies that include, but are not limited to exercise training, education, and behavior change, designed to improve the physical and psychological condition of people with chronic respiratory disease and to promote the long-term adherence to health-enhancing behaviors” [9]. Exercise training, specifically, is considered the core component of pulmonary rehabilitation [10].

Several systematic reviews have evaluated the effects of postoperative pulmonary rehabilitation interventions on lung cancer patients following surgery [11–17]. These reviews differ in their inclusion criteria and methodological quality. For instance, some reviews included various study designs, including observational studies, quasi-experimental trials, and randomized controlled trials [12, 17]. Furthermore, findings on the impact of exercise interventions on specific outcomes vary. For example, J. Wang et al. reported that postoperative exercise training did not significantly improve exercise capacity, as measured by the 6-min walk distance (6 MWD) (Weighted Mean Difference =—4.52 m, 95% confidence interval [CI]: −9.89—0.86 m) [16]. In contrast, Cavalheri et al. found a significant increase in 6 MWD among intervention groups compared to controls (Mean Difference [MD] = 57 m, 95% CI 34—80 m) [11].

The varied methodology and inconsistent findings complicate the interpretation of these findings, hindering the establishment of best practices for clinical application. Therefore, a comprehensive systematic review and meta-analysis is warranted. This systematic review and meta-analysis aimed to evaluate whether postoperative pulmonary rehabilitation exercise training effectively improves physical and psychological outcomes, such as QoL, exercise capacity, and respiratory function, in lung cancer patients following pulmonary resection. Furthermore, it is essential to identify effective intervention components to design future interventions. Therefore, this study also aims to identify the optimal intervention components influencing intervention effects. The findings may contribute to clinical applications and inform the development of postoperative pulmonary rehabilitation.

## Methods

### Study design

We present the systematic review and meta-analysis following the PRISMA and registered in the international prospective register of systematic reviews (PROSPERO) (No. CRD42024616958). The PRISMA 2020 Checklist is provided in Supplementary Material 1.

### Search strategies

Relevant literature was searched in five electronic databases: PubMed, CINAHL, Web of Science, Embase, and Scopus on September 20, 2024. The primary search terms of this review included “lung cancer” (“Lung Neoplasms” OR “lung tumor” OR “carcinoma non-small-cell lung” OR “carcinoma small-cell lung”) AND “pulmonary rehabilitation” (“respiratory rehabilitation”, OR “respiratory exercise”, OR “physical activity” OR “physical rehabilitation”). Additional relevant literature was identified by screening the reference lists of the articles included. The details of the search strategies are provided in Supplementary Material 2.

### Data selection process

A Covidence review manager was used to screen the retrieved articles. After removing duplicates, two independent reviewers screened the titles and abstracts to identify relevant articles. The full texts of the articles were assessed using the eligibility criteria. Any disagreements were resolved through discussions until a consensus was reached.

The inclusion criteria are as follows: a) lung cancer patients who had undergone lung cancer resection; b) the interventions in the rehabilitation group included pulmonary rehabilitation exercise training (e.g., breathing exercise techniques, walking, and strength) with or without any form of components of rehabilitation such as education, self-management; c) studies in which the control group received standard care (e.g., routine care, medicine guidance) without pulmonary rehabilitation, and active comparison conditions, such as different forms of exercise training; d) studies that reported at least one of the following parameters: physical outcomes, such as postoperative pulmonary complications, pulmonary function, and exercise capacity; psychological outcomes, such as QoL; e) randomized controlled trial published in English. The exclusion criteria are a) the study was published as an expert opinion, critical literature, abstract, or pilot study; b) the full text of the article was unavailable.

### Data extraction process

Data charting was performed by two independent reviewers. We extracted the following data using pre-formed tables, including the author, years of publication, country of study, study settings, population characteristics (sample size, mean age, and gender), intervention (type, frequency, duration, format, facilitators, and follow-up), and outcomes (data collection method and results).

### Study appraisal

The revised Cochrane risk-of-bias tool for randomized trials (RoB 2) was used to evaluate the methodological quality of the included studies [18]. Five aspects of the bias are assessed by this tool, including the randomization process, deviations from the intervention as intended, missing outcome data, outcome measurements, and the selective reporting of study results. Each criterion and overall bias are rated as “low risk of bias”, “some concerns”, or “high risk of bias”. Quality assessments were evaluated independently by two reviewers, and disagreements were resolved through discussions.

### Data synthesis and analysis

Data synthesis was performed using RevMan web, with findings visually shown in forest plots. Standardized mean difference (SMD) was calculated to compute effect sizes when outcomes were assessed by different tools. Furthermore, SMDs of 0.2, 0.5, and 0.8 were considered to be a small, medium, and large effect, respectively [19]. The changes in the value of the post and pre-intervention were pooled for the meta-analysis [19]. The I^2^ index was used to indicate heterogeneity. When I^2^ was less than 50%, a fixed-effects model was utilized; otherwise, a random-effects model was applied instead [20]. Publication bias was assessed if at least 10 studies reported the same outcomes, graphically reported by funnel plots, and tested using the Egger test using R software [21].

### Certainty of the evidence

The Grading of Recommendations Assessment, Development and Evaluation (GRADE) was used for examining the certainty of evidence through the Development and Evaluation profiler Guideline Development Tool (GRADEpro GDT). The evidence is classified into very low, low, moderate, and high levels of certainty from the five key assessment domains: risk of bias, inconsistency, indirectness, imprecision, and publication bias [19].

## Results

### Search results

**Fig. 1:**
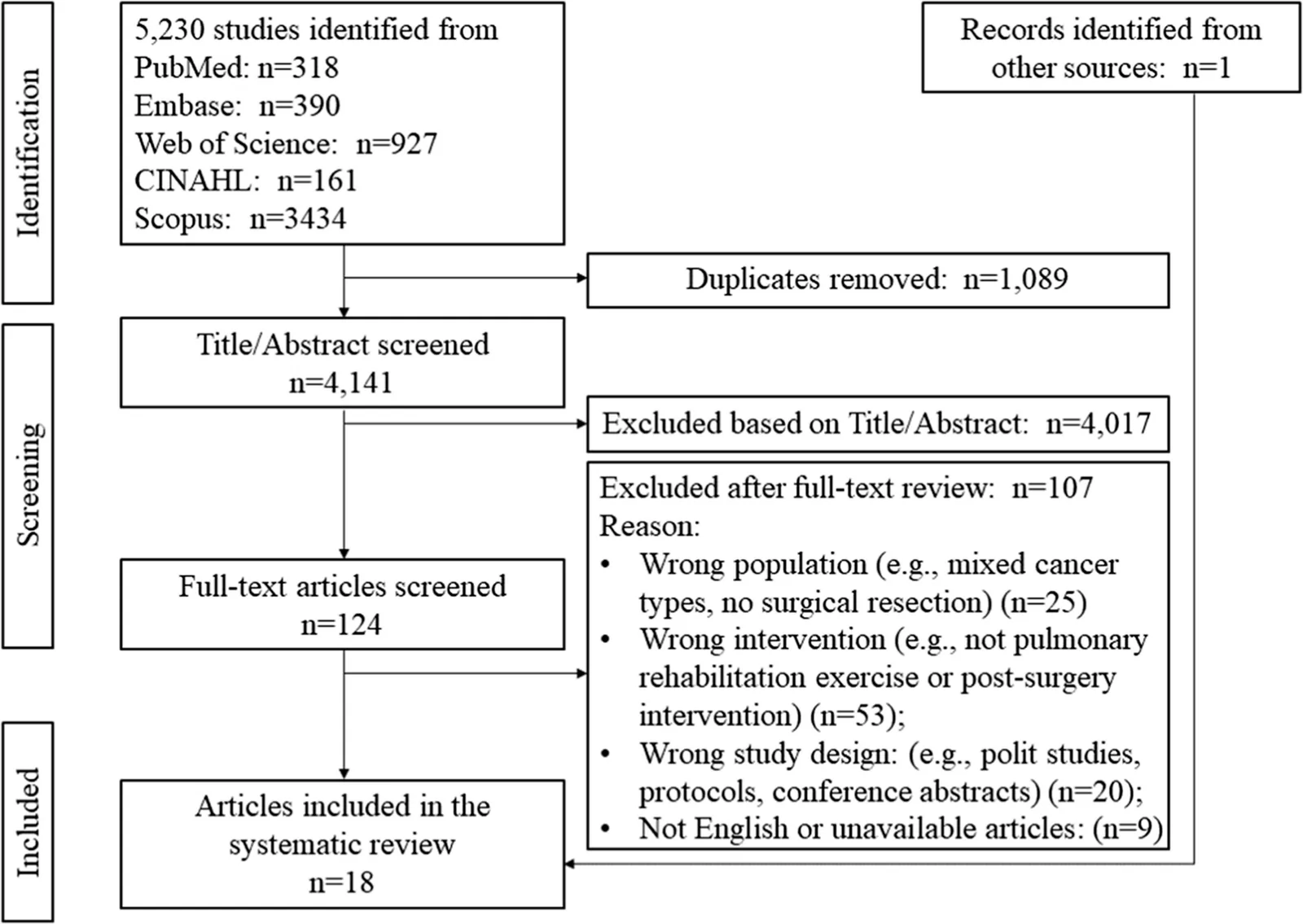
Flow of the literature search and screen

The systematic literature search initially retrieved 5,230 articles across five databases. After removing 1,089 duplicate records, the remaining 4,141 articles were screened for titles and abstracts, and then 4,017 articles were excluded. A full-text review of the remaining 124 articles was conducted, with 18 studies ultimately included in the analysis, including one additional record identified from other sources. The primary reasons for exclusion were wrong populations (n = 25), wrong interventions (n = 53), wrong study designs (n = 20), and articles unavailable or not published in English (n = 9). Among the included studies, two pairs of articles originated from the same studies but reported different outcomes [22–25]. The literature search and selection process is illustrated in Fig. 1.

### Characteristics of the included studies

The included studies were published between 2011 to 2024, representing a diverse geographical distribution including research from the United Kingdom [26, 27], Denmark [24, 25, 28], China [29–36], Norway [37], Sweden [22, 23], Portugal [38], and the Netherlands [39]. Study sample sizes varied, ranging from 32 to 235 participants. Participant age demographics were predominantly focused on older populations, with only two studies involving middle-aged participants (40–60 years) [30, 31] and one study including participants aged 18–70 [35].

Ten studies specifically included patients with non-small cell lung cancer [24–27, 31, 32, 35–37, 39]. Video-assisted thoracoscopic surgery (VATS) or open thoracotomy was utilized in the included studies. Seven studies were conducted at home [24, 25, 28, 29, 34, 38, 39], six studies at both home and hospital settings [22, 23, 26, 27, 33, 35], one at the hospital [30], one at a rehabilitation medicine center [36], one at fitness centers [37], one at elder health care unit [31], and one at a community center [32]. Detailed participant characteristics are presented in Table 1.

**Table 1:** Summary of the characteristics of participants

Author, year, country	Study setting	Lung Cancer type and stage	Operation type (n)	Allocation (n)	Mean age (Mean ± SD) Female (%)
Arbane 2011, United Kingdom	Hospital and home	NSCLC	Open thoracotomy 51 VATS 2	IG 27CG 26	IG 65.4; CG 62.6 Female total: 47.2%
Arbane 2014, United Kingdom	Hospital and home	NSCLC I, II, III, IV	Open thoracotomy: 90 VATS: 38	IG 64 CG 67	IG: 67 ± 11, Female, 55%CG: 68 ± 11, Female, 36%
Brocki 2014 Denmark	Home	Radically operated for lung cancer I, II, III	Open thoracotomy: 60 VATS: 18	IG 41 CG 37	IG: 64 ± 10, Female: 46.3% CG: 65 ± 9, Female: 35.1%
Cheng X 2022 China	Home	Lung cancer had undergone lobectomy	Not report	IG 29 CG 29	IG 62.8 ± 5.9, Female 48.3% CG 64.4 ± 7.1, Female: 55.2%
Edvardsen 2015 Norway	Fitness centres	NSCLC I–IV	Thoracotomy 51 VATS 10	IG 30 CG 31	IG = 64.4 ± 9.3, Female: 57% CG = 65.9 ± 8.5, Female: 52%
Ji 2023 China	Hospital	LC	Thoracoscopic or thoracotomy lobectomy	IG 32 CG 32	IG 42.92 ± 7.52, Female: 37.50% CG 45.31 ± 7.61, Female: 43.75%
Jiang 2020 China	Elder health care unit	NSCLC and had received surgery; experienced postoperative pain Excluded received chemo or radiotherapy within 6 months prior to lung surgery	VATS	IG 50 CG 50	IG = 59.30 ± 7.40, Female: 46.00% CG = 57.56 ± 11.23, Female: 42.00%
Liu, 2015 China	Community center	NSCLC stages I-IIIB, initial surgical resection was lobectomy (post-lobectomy lung cancer patients), ≥ 2 years after completion of surgical intervention	Not report	IG 16 CG 16	IG: 62.64 ± 8.35, Female: 43% CG: 60.46 ± 7.08, Female: 46%
Jonsson 2019 a Sweden	Hospital and home	Lung cancer; Previous thoracic surgery was not included	Thoracotomy 82 VATS 25	IG 54 CG 53	IG 68.7 ± 7.4, Female: 46.3% CG 68.4 ± 8.3, Female: 66%
Jonsson 2019 b Sweden	Hospital and home	Lung cancer; Previous thoracic surgery was not included	Thoracotomy 74 VATS 20	IG 50 CG 44	IG 69 ± 8, Female: 44% CG 68 ± 8, Female: 61%
Kendall 2020 Portugal	Home	Lung cancer	At any different pulmonary resections, except pneumonectomy	IMT 22 EMT 22 Combined 22 CG 22	IMT 62.31 ± 8.66, Female 23.1% EMT 60.00 ± 13.02, Female 38.5% Combined 64.06 ± 5.81, Female 50% CG: 63.42 ± 9.38, Female 36.8%
Liu J, 2021 China	Hospital and home	lung cancer IA -IIIA	VATS 54	IG 26 CG 28	IG 64.2 ± 5.9, Female: 53.8% CG 66.3 ± 7.9, Female: 64.3%
Qiu 2023 China	Home	Lung cancer, no mediastinal lymph node metastasis within stage II; Exclude receiving targeted therapy, chemotherapy, immunotherapy, and other treatments;	Not report	IG 40 CG 40	IG 62.76 ± 1.43, Female: 40% CG 62.18 ± 10.39, Female: 38.5%
Quist 2018 Denmark	Home	NSCLC stage I-IIIa assigned for curative intended surgery	Open: 39, VATS: 172	IG (early) 110 CG (late) 101	IG 66, Female: 58% CG 65, Female: 52%
Sommer 2020 Denmark	Home	NSCLC, scheduled for surgery with curative intention	VATS in both groups (early 80% and late 83%)	IG (early) 119 CG (late) 116	IG Median age 65 Female: 57.1% CG Median age: 65 Female: 53.4%
Stigt 2013 Netherlands	Home	Resectable patients with NSCLC	Over half of lobectomy or bilobectomy	IG 23 CG 26	IG: 63.6 ± 10.2, Female: 9% CG: 63.2 ± 10.3, Female: 27%
Xu 2024 China	Hospital and home	NSCLC, TNM stage IIIA and below (T0-3N0-2M0); completed the basic treatment of the current stage	Not report	IG 108 CG 108	Total age: 18–70
Yu 2024, China	Rehabilitation Medicine Center	NSCLC and COPD	Segmentectomy: 19 Lobectomy/bilobectomy: 54 Pneumonectomy: 1 VATS: 61 Thoracotomy: 23	CG 40 IG 44	CG: 69.6(4.8), Female 34.1% IG: 68.3(4.3); Female 30.0%

Abbreviations: CG: Control group; COPD: Chronic obstructive pulmonary disease; EMT: Expiratory muscle training; IG: Intervention group; IMT: Inspiratory muscle training; NSCLC: Non-small cell lung cancer; TNM: Tumor node metastasis classification; VATS: Video-assisted thoracoscopic surgery

### Results of quality appraisal

**Fig. 2:**
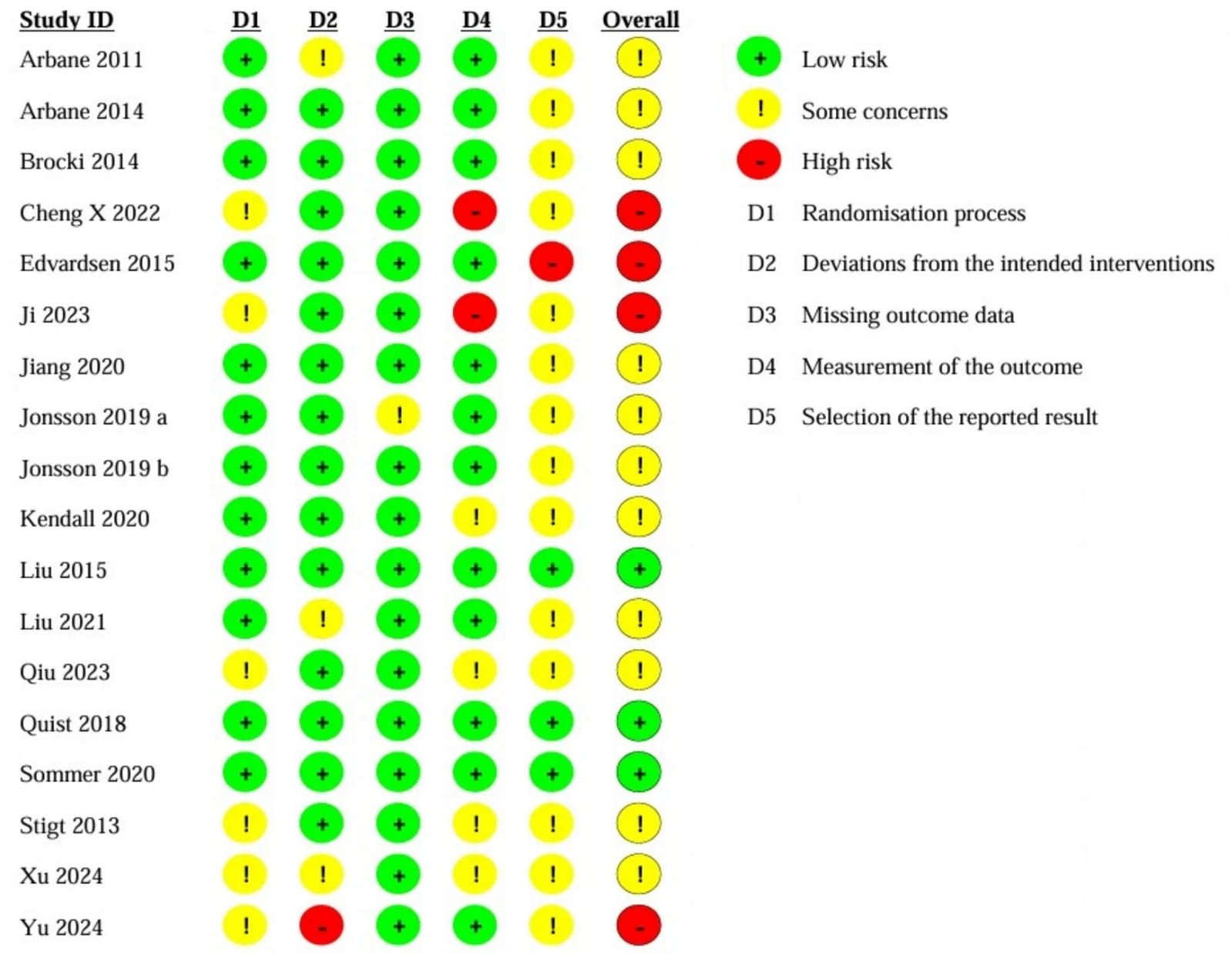
Critical appraisal of the methodological quality of the included studies

The results of the critical appraisal of the methodological quality of the included studies are presented in Fig. 2. Overall, only three studies were rated as having a “low risk of bias”, while four studies were rated as having a “high risk of bias”. Regarding the randomization process, six studies were rated as having “some concerns” mainly due to inadequate information provided about the randomization procedure and/or the status of allocation concealment. Most studies were rated as having a low risk of deviations from the intended interventions (78%) and in the domain of missing outcome data (94%). For the domain of “measurement of the outcomes”, six studies were rated as having some concerns or a high risk of bias. These concerns were mainly related to the failure of the studies’ authors to state whether the outcome assessors were aware of the intervention received by the participants or an open-label trial. Most of the studies (n = 15) were rated as either having some concerns or a high risk of bias in the selection of reported results, as a pre-specified analysis plan was not made available. This made it difficult to compare whether changes in methodologies used occurred between their proposed study plan and the actual procedures involved.

### Characteristics of the intervention

A summary of the pulmonary rehabilitation intervention is provided in Table 2. Five studies initiated the intervention on the first day post-surgery [22, 23, 26, 27, 30] and one study began on the day of chest tube removal [33]. Other studies started the intervention later, with eight studies commencing between one and seven weeks after surgery or discharge [24, 25, 28, 31, 35–37, 39].

**Table 2:** Summary of the pulmonary rehabilitation intervention

Author, year, country	Facilitator	Intervention format/Supervised/Session/frequency/intensity/duration	Control group	Follow-up	Main outcome (Measurement tool) Data collect timepoint	Conclusion
Arbane 2011, United Kingdom	Inpatient sessions were provided by research physiotherapy staff	Start: the first day after surgery Individualized In hospital (5 days): 1–5 postoperative day, supervised • Aerobic: mobility training, walking, as able, marching on the spot and recumbent bike exercises; 60%−80% HRmax for 5–10 min per exercise, 2/daily • Strength: seated leg raises with 2 lb ankle weights progressing to 4 lb Home: 12-week, unsupervised, monthly home visits and weekly telephone calls	Usual care Monthly telephone calls after discharge, providing education only	No follow-up	Pre-operatively, 5 day and 12 week post-operatively a) Quadriceps strength: magnetic stimulation b) Exercise tolerance: 6 MWD c) QoL: EORTC-QLQ-LC13 d) Length of stay, surgical intervention and POC	• Quadriceps strength over the 5-day in-patient period: IG > CG (p = 0.04) • 6 MWD: significant deterioration at 5 days post-operatively compared with pre-operatively, which returned to pre-operative levels by 12 weeks in both groups. No difference between IG and CG • QoL: unchanged over 12 weeks; • Length of stay, surgical intervention, and POC: No difference at any time point
Arbane 2014, United Kingdom	Not report	Start: the first day after surgery Individual In hospital: 1–5 postoperative day, supervised • Aerobic: cycle, 30 min/session, 50–60 r/min with the intensity increased steadily during the 3rd minute to achieve a maximum of 60% − 90% HR reserve and to 3 or 4 on BBS and 13–15 on RPE • Resistance: strength training, 10 maximum REP principle • Aimed to complete > 5 min on the 1 st postoperative day, increasing to a maximum duration of 30 min on the 5th postoperative day. Cool-down: ≥ 2 min unloaded pedaling until HR/oxygen saturation returned to within approximately 10 beats/minute of the resting value • Daily mobilization was encouraged Home: 4-week, daily, unsupervised, 30 continuous minutes home walking, pedometer. Telephoned weekly	usual care	No follow-up	pre-operatively, 5 days after surgery, and 4 weeks after surgery a) Physical activity: Actiwatch b) Quadriceps strength: c) Exercise tolerance: ISWT d) Qol: SF-36; EORTC QLQ-LC13 e) Length of stay, surgical intervention, and POC	Baseline to 4 weeks after surgery • Physical activity: no significant differences between groups • Quadriceps strength: no significant differences between groups • ISWT: no significant differences between groups • SF-36 or EORTC QLQ-LC13: no significant differences between groups • Length of stay, surgical intervention and POC: not statistically different between the groups
Brocki 2014 Denmark	Not report	Starting 3 weeks after surgery Supervised group-based, 1 h/week, 10 weeks Each session: • 15 min warming up (RPE 7–12), • 20 min aerobic exercise (intensity of 60–80% of work capacity RPE 11–12 (1–4 session) RPE 13–16 (5–10 session)) • 15 min muscle strength training (6–10 rep, 1–2 sets) • 10 min cooling down/relaxation • dyspnoea management Home exercise and usual postoperative nurse counselling same with control group	3 weeks after surgery, 1 h of individual instruction in exercise training Home exercising: Unsupervised, strength training at least 2/week, daily 30 min walk/bicycle ride, 11–12 RPE Usual postoperative nurse counselling: up to 3 individual 1-h sessions placed within 3 weeks to 4 months after surgery	12 months after surgery	3 weeks after surgery and after 4 and 12 months a) QoL: SF-36 b) Functional capacity: 6 MWD c) Lung function: spirometry, FEV1, FVC, dyspnoea	• A statistically significant effect (p = 0.01) after 4 months in the bodily pain domain of SF-36, a trend in favour of the intervention for role physical functioning (p = 0.07) and physical component summary (p = 0.06). At 12 months: CG presented overall slightly better measures • No effect of the intervention on 6 MWD, dyspnoea, or lung volumes at any time-point
Cheng X 2022	Not report	Pulmonary Rehabilitation Therapy 12 weeks • Exercise: 1) lower limb muscle movement: walking, running, stair climbing, treadmill exercise, power bicycle, etc. 2) upper limb muscle training: upper limb dynamometer, weightlifting, ball throwing. 3) whole physical exercise: housework including sweeping, and all kinds of traditional exercises such as swimming, rehabilitation exercise, and Qigong; 3–5 times/week, at least 2 to 3 months • Respiratory muscle training: inhale quickly and vigorously through the nose and abdomen at the same time, while slowly contracting the lips and exhaling and gradually contracting the abdomen • Nutritional therapy: nutritional support	Routine nursing after operation	No follow-up	Before and post-intervention a) Pulmonary Function: FEV1%, FEV1/FVC b) Exercise Ability: 6 MWD c) Dyspnoea: self-feeling score 0–40 d) Serum factors: CRP, IL-6, TNF-α e) Postoperative hospital stays, complications chest tube retention time	• IG: FEV1% and FEV1/FVC in the predicted value was significantly improved after intervention compared to before treatment • 6 MWD: IG > CG • Dyspnoea scores lower in IG than CG (p < 0.01) • IG > CG: average hospitalization time probability of pulmonary complications retention time of chest tube, CRP, IL-6 and TNF-α in serum
Edvardsen 2015 Norway	Highly qualified personal trainers and physiotherapists supervised each training session	Starting 5–7 weeks after surgery 60 min, 20 weeks, 2 weekly sessions individual; 1 weekly session group, if possible • High-intensity endurance and strength training, 3/week, supervised - Aerobic: HIIT, walking uphill on a treadmill, 80–95% HR_max_ - Progressive resistance training in 3 series of 6–12 RM of the leg press, leg extension, back extension, seat row, bicep curls and chest-and-shoulder press • Daily inspiratory muscle training, unsupervised	Usual care	No follow-up	Before surgery, 4–6 weeks after surgery and after intervention a) Exercise capacity: VO_2peak_ b) muscular strength: 1RM; concentric leg strength, handgrip c) daily physical functioning: chair stand test; maximum stair steps, static balance test d) Dyspnoea: QLQ-C30 e) Total muscle mass: dual-energy X-ray absorptiometry f) QoL: SF-36	IG had a greater increase in VO_2peak_ uptake (3.4 mL/kg/min between-group difference, p = 0.002), Tlco (5.2% predicted, p = 0.007), 1RM leg press (29.5 kg, p < 0.001), chair stand (2.1 times p < 0.001), stair run (4.3 steps, p = 0.002), total muscle mass (1.36 kg, p = 0.012), physical component (p = 0.006) and mental (p = 0.015) of QoL compared with the CG
Ji 2023 China	Rehabilitation therapists and nurses	ACBT Training with Watson’s Theory of Human Caring In hospital: 2/day, 6–8 cycle/session, supervised An ACBT cycle: 4 breath control exercises, 2 thoracic expansion exercises and 2 forced expiratory technique exercises ACBT should last 3 m after surgery Watson Care Implementation Plan • Health education: understand LC therapy and the importance of postoperative rehabilitation treatment • Nutritional support Nurses conduct intensive interventions once a week via telephone or WeChat for the 3 months following surgery	Routine care	No follow-up	Before and 1 month after the intervention a) Lung function: PEF, FEV1/FVC, VC, PaO2, OI and SaO2 b) Anxiety: SAS c) Depression: SDS d) QoL: QLQ-C30 e) Fatigue: Borg fatigue score f) Dyspnoea: Borg dyspnoea score g) Cardiopulmonary function: 6 MWD h) Post-operative complications: atelectasis, pulmonary infection, hypoxemia, respiratory failure, and pneumothorax	• Lung function levels (PEF, FEV1/FVC, VC, PaO2, OI and SaO2) in IG were significantly higher than in CG, while PaCO2 was significantly lower. After ACBT training, 6 MWD distance increased, and the maximum HR, Borg fatigue score, and Borg dyspnoea score in IG were lower than in CG • Clinical treatment, duration of drainage tube retention, total oxygen therapy, out-of-bed time, hospital stay, and VAS score in IG were all lower • QoL in IG were higher, and anxiety and depression were significantly lower • Incidence of postoperative complications was 6.25% in IG and 28.13% in CG
Jiang 2020 China	Tai Chi instructors	After one-month surgery 24-form Tai Chi 3 months, total 92 classes, daily 60 min (10-min warm-up, 40-min practice, and 10-min cool-down) Group, supervised	Routine nurse care	No follow-up	Before and after intervention a) Serum oxidative factors and inflammatory cytokines, b) KEAP1 and NRF2 in peripheral blood leukocytes, c) FEV1, FVC, SPO2, d) Pain scores	After intervention, TC significantly improve lung function by increasing the levels of KEAP1, NRF2, FVC, FEV1, and SPO2, and reducing pain scores (p < 0.05), increased the levels of antioxidant markers (SOD, catalase, GPx) and anti-inflammatory factor (IL-10), and reduced the levels of malondialdehyde and inflammatory markers (TNFα, IL-1 and IL-6) (p < 0.05)
Liu 2015 China	Expert Tai Chi practitioner	Tai Chi, supervised, 16 weeks, 3 times/week, 60 min/session: 15-min warm-up, 35-min practice, and 10-min cool-down (i.e., breathing with gentle stretching and tapping or self-massage) • First 8 weeks: emphasized static relaxation methods with music, breathing with up-down and open-close movements, and the mastery of single forms through multiple repetitions; • Later 8 weeks: 24-form Tai Chi based on Yang style and enhancing coordination of mind relaxation, breathing and locomotion	Hospital care	No follow-up	Before and after intervention a) PBMC proliferation and co-culture of PBMCs with the NSCLC cell line A549 were performed for proliferation and cell cytolysis assays b) Analysis of NKT cells, NK cells, and CD123 + and CD11c + dendritic cells	• After Tai Chi, cell proliferation increased significantly as compared with the control • PBMCs from the Tai Chi group also demonstrated enhanced cytolytic/oncolytic activity against A549 cells • Significant differences were also found in NK cell percentage at t = 16 weeks, post–pre changes of NKT and DC11c between groups
Jonsson 2019 a Sweden	Physiotherapists at the ward, all of whom were clinically educated to follow the intervention protocol	Physiotherapy intervention Preoperative treatment: Brief (5–10 min) individual preoperative physiotherapy information during admission day regarding the importance of postoperative mobilization Starting the first day after surgery • Postoperative treatment: 1–2 times/day, 10–30 min/session, during hospital stay (except Sunday): early mobilization, breathing exercises, and exercises for thoracic and shoulder range of motion; supervised • Postoperative information: physical activity, moderate level, 150 min/week • Individually adapted advice regarding breathing exercises at home: 10 deep breaths with PEP, repeated 3 times with 1-min rest between sets, 4–5 times/day, until they no longer experienced pain when taking deep breaths	standard care by the nursing staff	3 months postoperatively	preoperatively and 3 months postoperatively a) Physical capacity: 6 MWD b) physical activity: accelerometer, IPAQ c) lung function: FVC, FEV1 d) Dyspnoea: Modified Medical Research Council Dyspnoea Scale e) Pain: a numeric rating scale, 0–10	• No statistically significant differences between the groups: physical capacity, physical activity, spirometric values, or dyspnoea • IG increased the level of self-reported physical activity from preoperatively to 3 months postoperatively, while the patients in the CG did not
Jonsson 2019 b Sweden	Physiotherapists at the ward, all of whom were clinically educated to follow the intervention protocol	Same intervention with Jonsson 2019 a			Preoperatively and fourth day after surgery for patients still at the hospital a) Physical activity: accelerometer counts, steps per hour b) Physical capacity: 6 MWD, c) Lung function: FVC, FEV1 d) Dyspnoea: Modified Medical Research Council Dyspnoea Scale e) Pain: a numeric rating scale, 0–10	• IG showed an increased level of physical activity (accelerometer counts, steps per hour) during the first days after lung cancer surgery, as compared to an untreated control group • No significant differences between the groups regarding 6 MWD, spirometric values, pain, or dyspnoea
Kendall 2020 Portugal	Not report	After hospital discharge 8 weeks, six days/week, 15 min/session at a pace of one breathing effort and one resting cycle, supervised once a week • IMT group: Threshold IMT, first week 25% of MIP, increment of 5% load each week, until 60% of MIP • EMT group: Threshold PEP, same training load with IMT • Combined group: IMT + EMT, each 15 min, same load	Usual care	No follow-up	Day of admission and 8 weeks postoperatively a) Dyspnoea: MRC b) QoL: EQ-5D-3L c) Pulmonary and respiratory muscles function: FVC, FEV1, TI, PEF, TLC, DLCO, DLCO/VA, respiratory muscle function: MVV, MIP, MEP d) Physical fitness: 6MWD, e) Daily PA: accelerometry	• Significant treatment * time interactions were found for MIP (p = 0.014), sedentary PA (p = 0.003), light PA (p = 0.045) and total PA (p = 0.035). Improvements were observed for MIP in CombT (p = 0.001), IMT (p = 0.001), EMT (p = 0.050). SEDPA reduced in EMT (p = 0.001) and IMT (p = 0.006), while LIGPA increased in both groups (p = 0.001), Total PA (p = 0.005 and p = 0.001, respectively) • In UCare, CombT, and EMT, QoL improved only for Usual Activities
Liu J, 2021	Not report	Beginning on the day of chest tube removal to 6 weeks after discharge IMT and aerobic exercise, • IMT: A threshold load trainer, 30 breaths, 2/day, resistance load 30% of maximum inspiratory pressure, adjusted bi-weekly to 35% (2nd week), 40% (4th week), moderate-to intense IMT (6th week) - Flow-directed incentive spirometer (a device coaches patient to take a sustained maximum inspiration), 10–15 breaths/round, 3 rounds/day • Aerobic exercise: 60-min, daily - 250-mL water bottle for resisted upper limb uplifting, lower limb stepping exercise for 20 min/day, and a walking exercise (slow walking 5 min, brisk walking 2 min, and slow walking for 5 min) without a resistor - Intensity: RPE 11–13 at 50–70% exercise capacity • Telephonic interview on alternate days, and every 2 weeks during the patients’ follow-up visits to the clinic	Usual care: a standard perioperative pulmonary rehabilitation program that included smoking cessation and breathing control, upper and lower limb exercises, incentive spirometry, lung expansion therapy, and airway clearance therapy	12th week	Day of chest tube removal (baseline), 2, 6, and 12 weeks postoperatively a) Maximum inspiratory pressure (PImax), maximum expiratory pressure (PEmax) lung expansion volume, b) 6MWD	TG showed significant improvement in PImax at week 6, PEmax at week 2 and week 12, the lung expansion volume at week 2 and week 12, 6 MWD at week 2 and week 12
Qiu 2023 China	Nurse team	• Respiratory function exercise: pursed lip breathing; abdominal breathing; balloons can be used for blowing for 15 min 3–4 times a day; Postural nursing; Effective cough and expectoration exercise 3 months • Weekly telephone follow-up and online instant communication	Conventional nursing and health education	No follow-up	Before and post intervention a) Pulmonary function tests: SpO2, Vital capacity, MVV, FEV1, FEV1% pred, FEV1/FVC; b) blood gas analysis: PaO2 and PaCO2; c) respiratory symptoms: MDASI-LC; d) complications: atelectasis, pulmonary infection, incision infection, wound bleeding, and respiratory failure	Continuous nursing combined with respiratory exercise nursing could significantly accelerate the recovery of respiratory function, reduce the overall incidence rate of postoperative complications of lung cancer, and improve patients’ treatment compliance after lung cancer surgery
Quist 2018 Denmark	Trained physiotherapists and cancer nurse specialists	Early intervention: 2 weeks after surgery 12-week exercise: supervised, 2/week, 60 min/sessions, total 24 sessions • individually strength: 25 min, leg press, chest press, leg extension, pull to chest, and pull down (upper body) • Group-based cardiovascular exercise: - Warm-up (5 min) aimed at reaching 85% HRmax, followed by a short rest (1 min) - HIIT: 25 min on an ergometer. In each interval (1–2 min), aimed at reaching 85%−100% HRmax, followed by a short rest (1 min). The HIIT was followed by 2 min cool down • All exercise sessions included supervised breathing exercises combined with stretching and tension-release techniques (5 min) • 3 individual counselling sessions • 3 group-based lessons in health-promoting behavior	Late intervention: - Usual care (2–14 weeks after surgery) - 14 weeks after surgery	52 weeks follow-up following surgery	Baseline (day before surgery), after 14 weeks (end of early rehabilitation/beginning of late rehabilitation), 26 weeks (end of late rehabilitation) and 52 weeks follow-up a) VO2peak b) Fatigue: EORTC QLQ C30 LC13	• A significant difference from baseline to 14 weeks between groups (p = 0.0018). a significant difference from 14 to 26 weeks between the two groups (p < 0.001) • No significant differences in QOL but a significant difference between ERG and LRG from baseline to 14 weeks in fatigue level in favour of early intervention group
Sommer 2020 Denmark		Early intervention: 2 weeks after surgery 12-week exercise: supervised, 2/week, 60 min/sessions, total 24 sessions •Individually strength: 25 min, leg press, chest press, leg extension, pull to chest, and pull down (upper body) •Group-based cardiovascular exercise: -Warm-up (5 min) aimed at reaching 85% HRmax, followed by a short rest (1 min) -HIIT: 25 min on an ergometer. In each interval (1–2 min), aimed at reaching 85%−100% HRmax, followed by a short rest (1 min). The HIIT was followed by 2 min cool down •All exercise sessions included supervised breathing exercises combined with stretching and tension-release techniques (5 min) •3 individual counselling sessions •3 group-based lessons in health-promoting behavior	Late intervention: - Usual care (2–14 weeks after surgery) - 14 weeks after surgery	52 weeks after surgery	Baseline, 14 weeks, 26 weeks and 52 weeks after surgery HRQoL: FACT-L and FACT-G	• In IG, HRQoL showed a continuous improvement up to 26 weeks after which HRQoL decreased after further 26 weeks without structured intervention • In CG a non-significant deterioration was detected over the first 14 weeks after surgery. After participation in the 12 weeks rehabilitation program, an increase in HRQoL was seen, without reaching the same level as the early group
Stigt 2013 Netherlands	Exercise training by physiotherapists and scheduled visits to a specialized pain team and medical social worker	Four weeks after discharge 12-week 2/week, 60 min/session Aerobic (cycling): 60%−80% of peak cycling load Resistance training Supervised Not report group or individual	usual care: routine outpatient appointments, scheduled 1, 3, 6, and 12 months after discharge. Pain medication was prescribed by their pulmonologist or general practitioner Referrals to physiotherapy or social work occurred on a personalized base	1-year follow-up	Before thoracotomy, 1, 3, 6, and 12 months after discharge a) QOL: SGRQ; SF-36 b) Pain: MPQ-DLV c) PFT and exercise capacity (6MWD): before surgery and 3 m after discharge	• QOL was not significantly different between groups, the IG reported more pain after 3 and 6 months and more limitations because of physical problems after 3 months • IG: 6MWD improved by 35 m from preoperative baseline, CG decline by 59 m(p = 0.024)
Xu 2024 China	Baduanjin qigong by rehabilitation technicians from the School of Sports rehabilitation of Beijing Sport University in the first week	After completing basic treatment during hospitalization, Baduanjin qigong by rehabilitation technicians from the School of Sports rehabilitation of Beijing Sport University in the first week Eight postures Baduanjin qigong supervised in the first week One week later, Baduanjin Qigong was at home 30 min, 2/day, 4 days/week, 12 weeks Health education: understanding the importance of post-operative pulmonary rehabilitation to improve patients' compliance, education and urging them to quit smoking, avoid colds and infections	• Routine breathing training: lip-constricted breathing (2 times/d, 10 min/time) and simple deep inspiratory trainer (2 times/d, 15 min/time), encouraged to participate in competent physical activities • Health education same as intervention group	No-follow up	Before and after intervention a) Lung function: FVC%, FEV1% b) Quality of life: 6MWD, Piper Fatigue Scale, and Borg dyspnoea score	lung function, 6MWD, Piper fatigue Scale score and Borg score were significantly better in the two groups after intervention (P < 0.05) No significant differences in FVC%, 6MWD and Borg score between the two groups (P > 0.05), whereas FEV1% and Piper fatigue Scale scores in the IG were better than that in CG (P < 0.05)
Yu, 2024, China	Not report	Start after one-week of the surgery • Aerobic training: cycle ergometers • 30 min each session, twice daily, 6 days weekly • 20% HR reserve at the beginning and gradually increased to 60% to 70% HR reserve • 2 weeks	Standard postoperative rehabilitation for 1 week in the surgery department: early mobilization, cough, and deep-breath technique teaching, supplemental oxygen therapy and nebulization After the first week being transferred to the Rehabilitation Medicine Center: usual care, such as oxygen therapy, nebulization	No follow-up	Baseline:3 days before surgery; Endpoint: 1 day after exercise program a) Physical capacity: CPET (peak VO_2_), 6MWD b) Overall lung function: FVC, FEV1 c) daily functional ability: Daily walking step d) QoL: QLQ-C30, higher, better	Drop out 14.3% • Peak VO_2_, 6MWD: IG > CG, P < 0.05 • FVC, FEV1: IG > CG, P > 0.05 • daily walking step: IG > CG, P < 0.05 • QoL: decline in IG smaller, IG VS CG, P > 0.05

Abbreviations: 6 MWD: 6-min walk distance; ACBT: Active Cycle of Breathing Technique; BBS: Borg Breathlessness Scale; CG: control group; CPET: Cardiopulmonary exercise test; DLCO: diffusing capacity of the lung for carbon monoxide, DLCO/VA: diffusing capacity of the lung for carbon monoxide per unit of alveolar volume; EORTC QLQ-C30: European Organisation for Research and Treatment of Cancer, Quality of Life Questionnaire Core-30; EORTC QLQ-LC13: European Organisation for Research and Treatment of Cancer, Quality of Life Questionnaire—Lung Cancer; EQ-5D-3L: EuroQol five-dimension three-level questionnaire; FACT-G: Functional Assessment of Cancer Therapy – General; FACT-L: Functional Assessment of Cancer Therapy- Lung scale; FEV1: Forced expiratory volume in the first second; FEV1/FVC: Forced expiratory volume in 1 s/forced vital capacity ratio; FVC: Force vital capacity; GPx: glutathione peroxidases; HIIT: High-intensity interval exercise; HR: Heart rate; HRQoL: Health-related quality of life; IG: Intervention group; IL-1: interleukin-1; IL-6: interleukin-6; IL-10: interleukin-10; IMT: inspiratory muscle training, ISWT: Incremental Shuttle Walk Distance; MDASI: modified lung cancer module MD Anderson Symptom Assessment Scale; MIP: maximal inspirator pressure; MEP: maximal expiratory pressure; MPQ-DLV: McGill Pain Questionnaire; MVV: maximum voluntary ventilation; MVV: maximum voluntary ventilation, MRC: Medical Research Council Dyspnoea Questionnaire; NK: natural killer; OI: oxygenation index; PBMC: Peripheral blood mononuclear cells; PaCO2: partial pressure of carbon monoxide; PaO2: partial pressure of oxygen dissolved in blood; PEF: peak expiratory flow; PFT: pulmonary function test; PEmax: maximum expiratory pressure; PImax: Maximum inspiratory pressure; POC: post-operative complications; QoL: Quality of life: RPE: Rating of Perceived Exertion; RM: repetition maximum; SaO2: oxygen saturation; SAS: Self-Rating Anxiety Scale, SDS: Self-rating Depression Scale; SF-36: Medical Outcomes Study Short-Form 36; SGRQ: St. George’s respiratory questionnaire; SOD: superoxidase dismutase; SpO2: spirometry and peripheral oxygen saturation; TI: Tiffeneau index; Tlco: carbon monoxide transfer factor; TLC: total lung capacity; TNFα: tumor necrosis factor α; VAS: visual analog score; VC: vital capacity; VO_2peak_: peak oxygen uptake

#### Format

Fifeen studies focused on exercise intervention. Four studies exclusively targeted aerobic exercise [31, 32, 35, 36]. Four studies integrated aerobic and strength training [26–28, 39]. Seven studies combined exercise with breathing exercises. Three studies employed a combination of aerobic exercise, resistance training, and breathing exercise [24, 25, 37]; other combinations involved whole-body physical activity with breathing exercise [22, 23, 29]; and aerobic exercise coupled with inspiratory muscle training (IMT) [33].

Respiratory-focused interventions were examined in three studies. One involved breathing exercises incorporating IMT and expiratory muscle training (EMT) [38], while another incorporated respiratory function exercises such as pursed lip breathing, abdominal breathing, and balloon techniques [34]. Additionally, one study involved the active cycle of breathing technique [30].

The primary forms of aerobic exercise included cycling, walking, and treadmill activities. The main forms of resistance training included exercises targeting major muscle groups, such as leg raises, presses, extensions, back exercises, rowing, bicep curls, and chest and shoulder presses. Breathing training encompassed respiratory muscle training, such as threshold-based IMT and EMT with positive expiratory pressure (PEP), as well as breathing techniques such as pursed-lip and abdominal breathing.

#### Intensity

The moderate intensity of aerobic exercise, ranging from a maximum of 60–90% of heart rate reserve, was investigated in eight studies [22, 23, 26–28, 33, 36, 39]. Three studies employed high-intensity interval training (HIIT) at a maximum of 80–100% heart rate reserve [24, 25, 37]. Strength training intensity was reported as specifying 6–10 repetitions [26, 28] and 6–12 repetitions maximum [37]. Breathing exercise intensities varied, including protocols of 10 deep breaths with PEP [22, 23], and progressively increasing inspiratory muscle training from 30% to moderate-to-intense levels using a threshold load trainer [33]. Some studies implemented threshold inspiratory and expiratory muscle training, gradually increasing from 25 to 60% of maximal inspiratory pressure (MIP) or maximal expiratory pressure (MEP) [38].

#### Duration and frequency

The duration of each intervention session ranged from 10 to 30 min in hospital settings and 30 to 60 min at home. The overall intervention duration spanned from 2 to 20 weeks. Seven studies included follow-up periods ranging from 12 weeks to 12 months. The frequency of aerobic or resistance exercise interventions varied from one to eight times per week. Breathing training sessions were conducted 6 times per week, as frequently as 2–5 times a day.

#### Group or individualized

Two studies in group-based intervention [28, 31], while five studies implemented individualized intervention [22, 23, 26, 27, 37]. Additionally, two articles from the same study combined group-based cardiovascular exercise with individualized strength training [24, 25].

#### Supervision

Supervised interventions were conducted in various settings. Five studies provided supervised interventions in hospital settings [22, 23, 26, 27, 30]. Six studies implemented home-based supervision [24, 25, 28, 35, 38, 39]. Others provided supervision in community settings, such as fitness centers [37], elder health care centers [31], and community centers [32].

### Control group

In 15 studies, the control group received standard care. One study used unsupervised home exercise consisting of aerobic and strength training in the control group [28]. In two studies, the control group initially received usual care for the first 12 weeks, followed by a late intervention [24, 25].

### Main outcome

#### Lung function

##### Forced vital capacity (FVC)

**Fig. 3:**
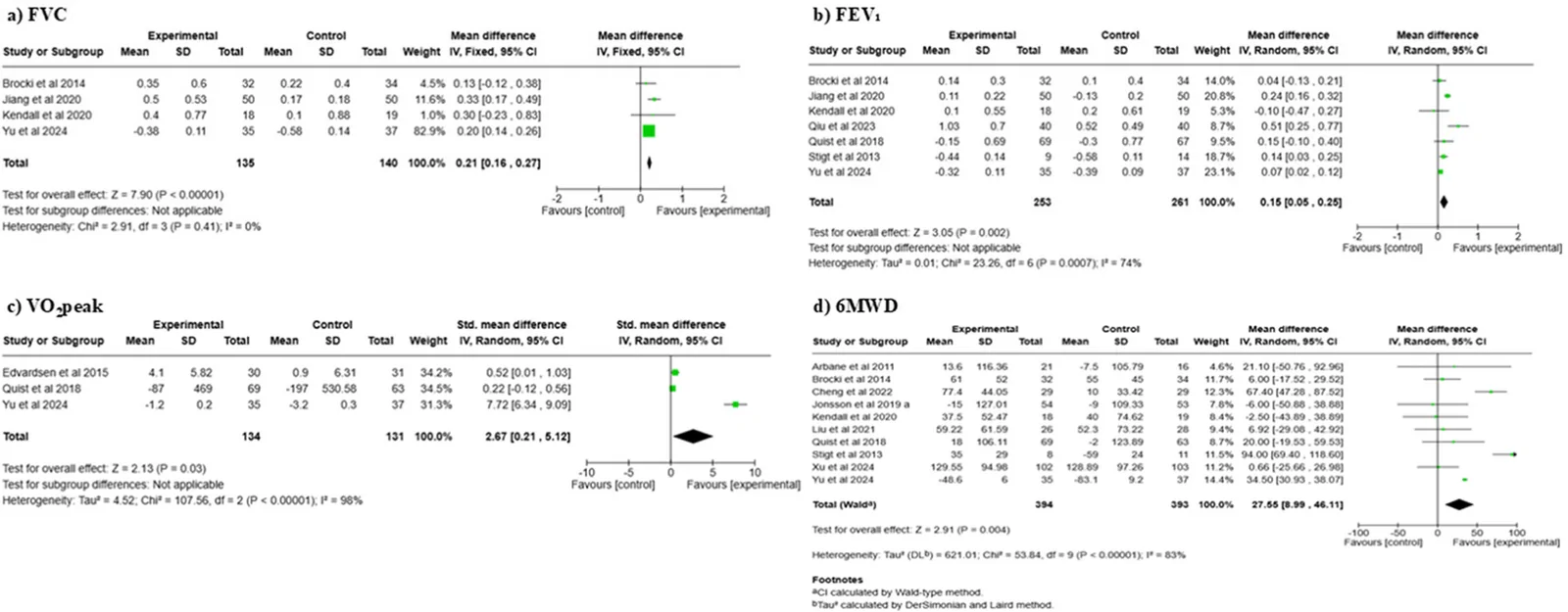
Forest plot of changes in FVC, FEV₁, VO2peak values and 6MWD

FVC was reported in four studies involving 275 participants [28, 31, 36, 38]. There were three breathing exercise groups, including the IMT, EMT, and combination, in Kendall et al.’s study; we included the combing group data in the meta-analysis. Using a fixed-effects model and MD for effect estimation, the results suggested a significant improvement in the intervention compared to the control groups (MD = 0.21; 95% CI [0.16, 0.27]; P < 0.001, I^2^ = 0%). The FVC results are presented in Fig. 3a. Sensitivity analyses, conducted by excluding studies one by one, confirmed the consistency of the results. The level of certainty of the evidence was moderate. Details of the level of certainty are presented in Table 3.

**Table 3:** Summary of findings

Outcomes	Anticipated absolute effects^*^ (95% CI)		Relative effect (95% CI)	№ of participants (studies)	Certainty of the evidence (GRADE)	Comments
	Risk with [Control]	Risk with [Intervention]				
FVC	The mean FVC was **0**	MD **0.21 higher** (0.16 higher to 0.27 higher)	-	275 (4 RCTs)	⨁⨁⨁◯ Moderate^a^	
FEV1	The mean FEV1 was **0**	MD **0.15 higher** (0.05 higher to 0.25 higher)	-	514 (7 RCTs)	⨁⨁◯◯ Low^a,b^	
VO_2 peak_	-	SMD **2.67 SD higher** (0.21 higher to 5.12 higher)	-	265 (3 RCTs)	⨁◯◯◯ Very low^a,b,c^	
6 MWD	The mean 6 MWD was **0**	MD **27.55 higher** (8.99 higher to 46.11 higher)	-	787 (10 RCTs)	⨁⨁⨁◯ Moderate^a,b,d^	
Quality of life-physical domain	The mean quality of life-physical domain was **0**	MD **3.72 higher** (1.15 higher to 6.29 higher)	-	308 (4 RCTs)	⨁⨁⨁◯ Moderate^a,c^	
Quality of life-mental domain	The mean quality of life-mental domain was **0**	MD **2.41 higher** (3.46 lower to 8.29 higher)	-	308 (4 RCTs)	⨁◯◯◯ Very low^a,b,c^	
Pain	-	SMD **0.37 SD lower** (1.28 lower to 0.51 higher)	-	264 (3 RCTs)	⨁⨁⨁◯ Moderate^b^	
Dynopnea	-	SMD **0.35 SD lower** (0.77 lower to 0.08 higher)	-	550 (6 RCTs)	⨁◯◯◯ Very low^a,b,c^	

*The risk in the intervention group (and its 95% confidence interval) is based on the assumed risk in the comparison group and the **relative effect** of the intervention (and its 95% CI).

***CI*** confidence interval; *MD* mean difference; *SMD* standardised mean difference

**GRADE Working Group grades of evidence**

**High certainty:** we are very confident that the true effect lies close to that of the estimate of the effect

**Moderate certainty:** we are moderately confident in the effect estimate: the true effect is likely to be close to the estimate of the effect, but there is a possibility that it is substantially different

**Low certainty:** our confidence in the effect estimate is limited: the true effect may be substantially different from the estimate of the effect

**Very low certainty:** we have very little confidence in the effect estimate: the true effect is likely to be substantially different from the estimate of effect

**Explanations**

^a^Some studies rated as having a high risk of bias

^b^Some level of inconsistency across results of studies

^c^Some studies rated as having a high risk of reporting bias

^d^Large effect, and mean difference reached clinical important difference

##### Forced expiratory volume in one second (FEV1)

FEV1 was reported in seven studies involving 514 participants [24, 28, 31, 34, 36, 38, 39]. Due to substantial heterogeneity (I^2^ = 74%), a random-effects model was used. The results showed a significant improvement in the intervention group compared to the control group (MD = 0.15; 95% CI [0.05, 0.25]; P = 0.002). Figure 3b presents the effects of the interventions on FEV₁. Sensitivity analyses confirmed the consistency of the results. The level of certainty of the evidence was low.

#### Physical capacity

##### Peak oxygen uptake (VO₂peak)

Figure 3c presents the impact of the interventions on VO₂peak. VO₂peak was reported in three studies with 265 participants [24, 36, 37]. One study measured VO₂peak in mL O₂/min, while the other two used mL O₂/min/kg, necessitating the use of SMD for data analysis. Due to high heterogeneity (I^2^ = 98%), a random-effects model was applied. The results indicated a significant improvement in the intervention group compared to the control group (SMD = 2.67; 95% CI [0.21, 5.12]; P = 0.03). The level of certainty of the evidence was very low.

##### 6 MWD

6 MWD was reported in 10 studies involving 787 participants [22, 24, 27–29, 33, 35, 36, 38, 39]. With substantial heterogeneity (I^2^ = 83%), a random-effects model was used for effect estimation. The results revealed a significant improvement in the intervention group compared to the control group (MD = 27.55; 95% CI [8.99, 46.11]; P = 0.004). Figure 3d presents the impact of the interventions on 6 MWD. The level of certainty of the evidence was moderate.

#### QoL

##### Physical Domain

**Fig. 4:**
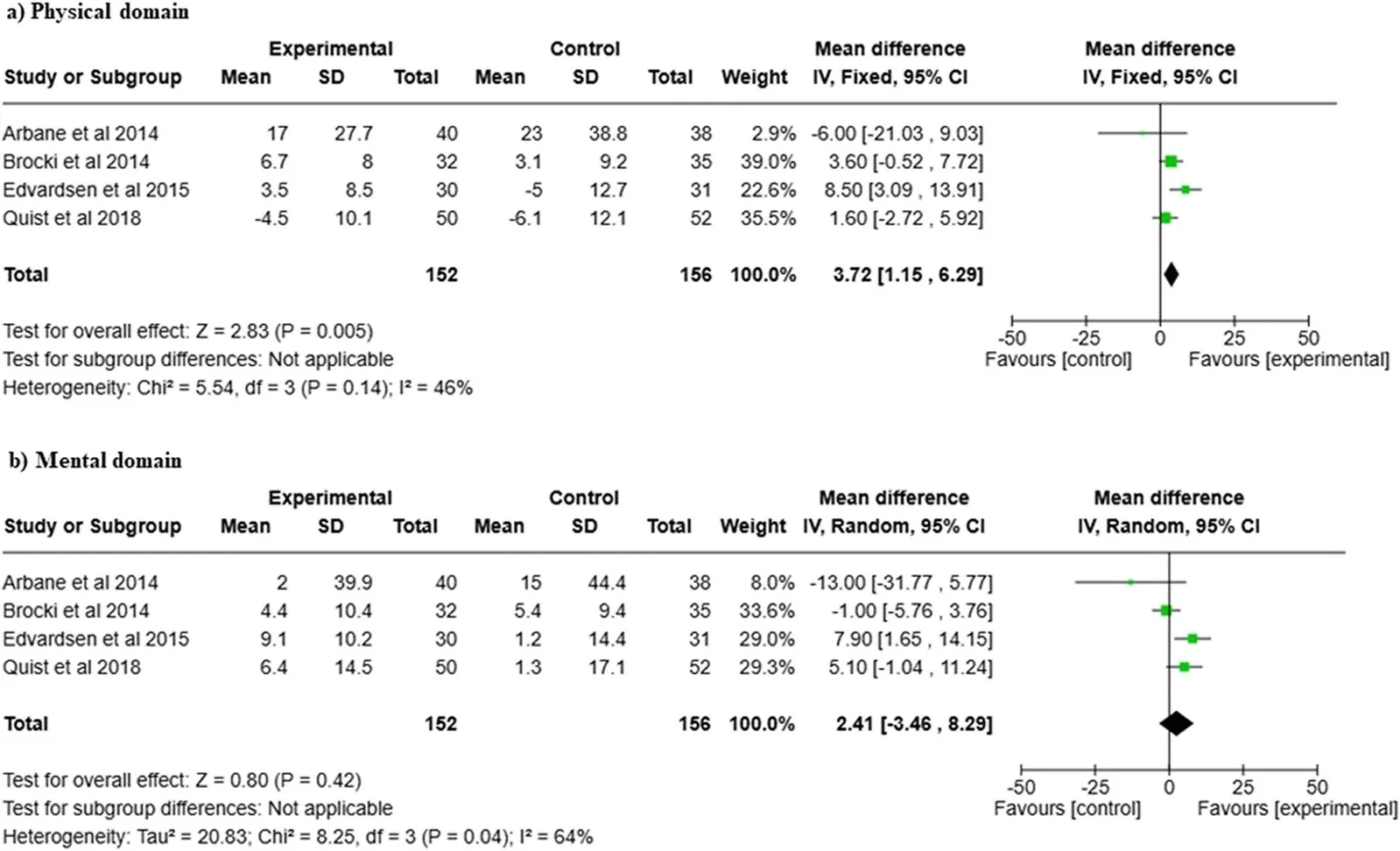
Forest plot of changes in the physical and mental domain of quality of life

Four studies with 308 participants reported on the physical domain of QoL, measured using the 36-Item Short Form Survey (SF-36) scale [24, 26, 28, 37]. A fixed-effects model was applied due to moderate heterogeneity (I^2^ = 46%). The results showed a significant improvement in the intervention group compared to the control group (MD = 3.72; 95% CI [1.15, 6.29]; P = 0.005). The effects of the interventions on the physical domain of QoL are shown in Fig. 4a. The level of certainty of the evidence was moderate.

##### Mental Domain

The same four studies also reported on the mental domain of QoL [24, 26, 28, 37]. Due to higher heterogeneity (I^2^ = 64%), a random-effects model was used. The pooled analysis did not show a statistically significant difference between the intervention and control groups. Figure 4b displays the impact of the interventions on the mental domain of QoL. The level of certainty of the evidence was very low.

### Pain

Pain scores were reported in three studies involving 264 participants [24, 31, 38]. Different measurements, such as the Prince-Henry Score, the pain domain of the EuroQol five-dimension questionnaire (EQ-5D), and the European Organisation for Research and Treatment of Cancer questionnaire (EORTC), were used, necessitating the use of SMD for analysis. The pooled results showed no significant difference between the intervention and control groups (Supplementary Fig. 1). The level of certainty of the evidence was moderate.

### Dyspnoea

Dyspnoea was assessed in six studies with 550 participants using various scales, including the Borg Scale, self-feeling score of dyspnoea, dyspnoea domain of EORTC Quality of Life Questionnaire Core-30 (QLQ-C30), and the Medical Research Council Dyspnoea Scale [24, 28, 29, 35, 37, 38]. The pooled analysis did not show a significant difference between the intervention and control groups (Supplementary Fig. 2). The level of certainty of the evidence was very low.

### Other outcomes

No significant differences were observed in the length of hospital stay and post-operative complications between the intervention and control groups [26, 27]. However, studies by Cheng, Ji, and Yan, and Qiu et al. [29, 30, 34] reported better outcomes in the intervention group compared to the control group. The intervention group demonstrated significantly better outcomes in serum inflammatory markers, such as C-reactive protein, interleukin-6, and tumor necrosis factor-alpha [29].

### Subgroup analysis

#### Subgroup analysis of FVC

For FVC, subgroup analyses revealed that exercise-based interventions had a significant effect, with an MD of 0.21 (95% [CI]: 0.16–0.27, P < 0.001), as shown in Supplementary Fig. 3. Further exploration indicated that aerobic exercise specifically yielded significant improvements (MD = 0.22, 95% CI: 0.16–0.27, P < 0.001) (Supplementary Fig. 4). Additionally, interventions lasting 12 weeks or longer demonstrated greater efficacy (MD = 0.33, 95% CI: 0.17–0.49, P < 0.001) (Supplementary Fig. 5). Both supervised (MD = 0.28, 95% CI: 0.15–0.40, P < 0.0001) and unsupervised interventions (MD = 0.20, 95% CI: 0.14–0.26, P < 0.00001) showed improvements in FVC (Supplementary Fig. 6). Since only two studies reported the intensity of the exercise intervention, subgroup analysis by intensity was not performed. However, given that only four studies were included in this analysis, the results should be interpreted with caution.

#### Subgroup analysis of FEV1

Regarding FEV1, subgroup analysis similarly highlighted the positive effect of exercise interventions (MD = 0.13, 95% CI: 0.04–0.22, P = 0.003), as shown in Supplementary Fig. 7. Further subgroup analysis showed that aerobic and resistance exercises produced notable improvements (MD = 0.12, 95% CI: 0.03–0.20, P = 0.008), Supplementary Fig. 8. Moreover, interventions with a duration of at least 12 weeks showed an enhanced impact on FEV1 (MD = 0.23, 95% CI: 0.11–0.34, P < 0.001), Supplementary Fig. 9. Nevertheless, the findings for the breathing subgroup were based on only two studies and require cautious interpretation. Interventions with supervision demonstrated improvement in FEV1 (MD = 0.14, 95% CI: 0.05–0.24, P = 0.002) (Supplementary Fig. 10). Four studies provided the intensity of the exercise intervention; subgroup analysis showed that moderate-intensity exercise improved FEV1 (MD = 0.08, 95% CI: 0.04–0.12, P = 0.0002) (Supplementary Fig. 11). However, due to the small number of studies and heterogeneity, these findings should be interpreted with caution.

#### Subgroup analysis of 6 MWD

For 6 MWD, exercise interventions resulted in a significant improvement (MD = 30.92, 95% CI: 6.04–55.80, *P* = 0.01), Supplementary Fig. 12. However, further analysis did not reveal significant differences between various exercise formats. Longer intervention durations (≥ 12 weeks) were associated with greater improvements (MD = 43.33, 95% CI: 5.22–81.43, P = 0.03), Supplementary Fig. 13. Interventions without supervision demonstrated improvement in 6 MWD (MD = 39.16, 95% CI: 12.68–65.63, P = 0.004) (Supplementary Fig. 14). Seven studies provided the intensity of the exercise intervention; subgroup analysis showed that moderate-intensity exercise was associated with improved 6 MWD (MD = 29.19, 95% CI: 2.81–55.57, P = 0.03) (Supplementary Fig. 15). Nonetheless, these findings are based on a small number of studies with heterogeneity and should be interpreted with caution.

#### Subgroup analysis of QoL

QoL outcomes were also examined. Exercise combined with breathing interventions showed significant benefits in both physical (MD = 8.50, 95% CI: 3.09–13.91, *P* = 0.002) (Supplementary Fig. 16) and mental domains (MD = 7.90, 95% CI: 1.65–14.15, *P* = 0.01) (Supplementary Fig. 17). Subgroup analysis based on intervention duration further indicated that programs lasting at least 12 weeks yielded significant improvements in physical (MD = 4.29, 95% CI: 0.91–7.66, *P* = 0.01) (Supplementary Fig. 18) and mental (MD = 6.48, 95% CI: 2.10–10.86, *P* = 0.004) QoL (Supplementary Fig. 19). Subgroup analysis showed that HIIT exercise was associated with improvement in the physical domain (MD = 4.29, 95% CI: 0.91–7.66, P = 0.01) (Supplementary Fig. 20) and mental domain (MD = 6.48, 95% CI: 2.10–10.86, P = 0.004) (Supplementary Fig. 21). These results, however, were derived from only four studies and should be interpreted cautiously.

#### Subgroup analysis of dyspnoea

For dyspnoea, exercise combined with breathing interventions significantly reduced symptoms (MD = −1.03, 95% CI: −1.71 to −0.35, *P* = 0.003) (Supplementary Fig. 22). Interventions lasting at least 12 weeks were more effective, with reductions in dyspnoea scores (MD = −0.57, 95% CI: −1.09 to −0.04, *P* = 0.03) (Supplementary Fig. 23). Nonetheless, the breathing subgroup included only two studies, warranting careful consideration of the findings. Unsupervised interventions demonstrated improvement in dyspnoea (SMD = −1.39, 95% CI: −1.96 to −0.81, P < 0.00001) (Supplementary Fig. 24). Three studies provided the intensity of the exercise intervention; subgroup analysis showed that HIIT exercise improved dyspnoea (SMD = −0.47, 95% CI: −0.78 to −0.16, P = 0.003) (Supplementary Fig. 25). However, both the unsupervised and moderate intensity subgroups included only one study each, and the observed heterogeneity warrants careful interpretation of these findings.

### Publication bias

**Fig. 5:**
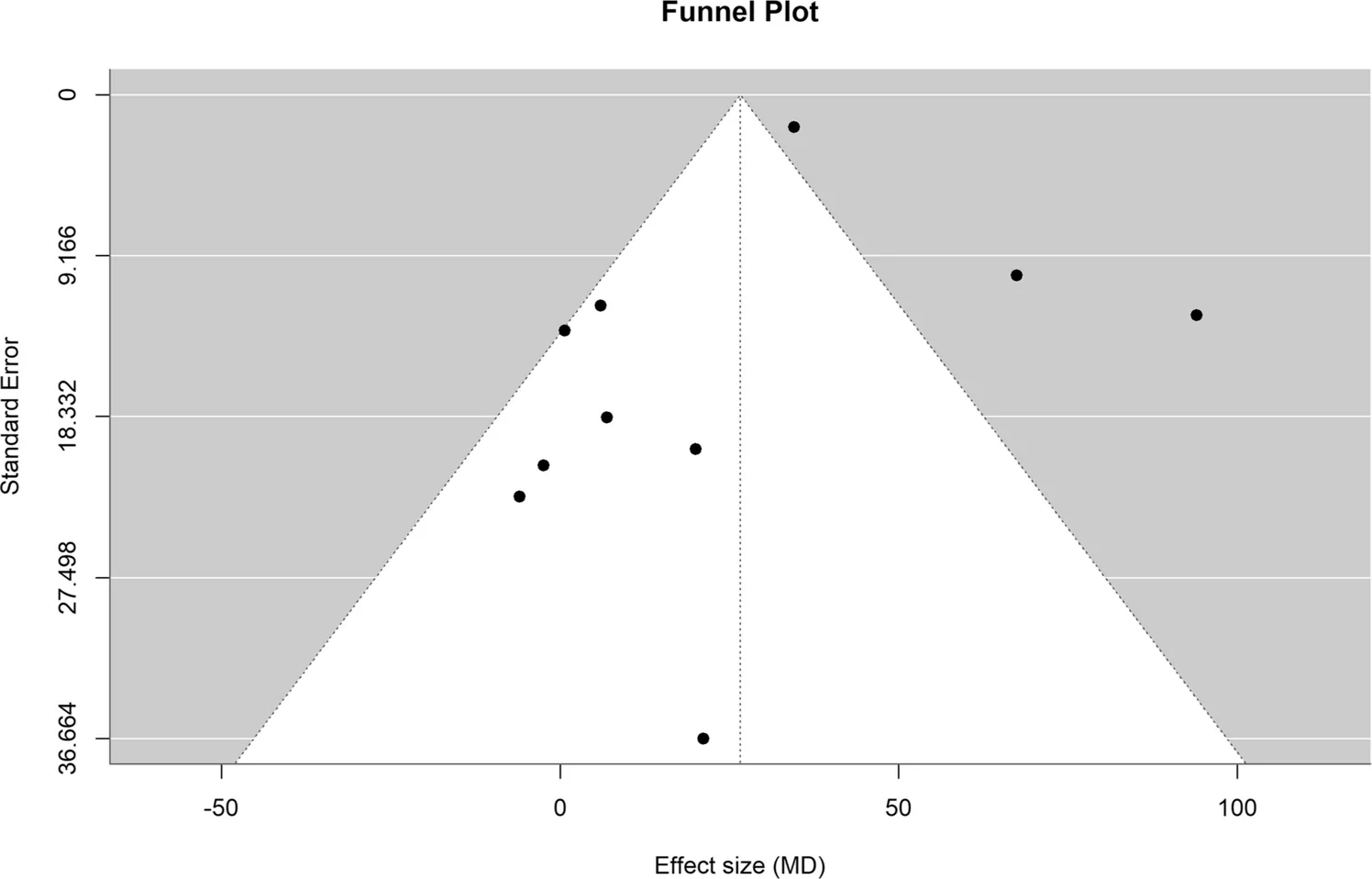
Funnel plot for publication bias assessment

Publication bias was assessed using a funnel plot (Fig. 5), which displayed reasonable symmetry of data points, suggesting an absence of substantial bias. This was supported by Egger’s test (z = -1.09 *P* = 0.275), indicating that the results of the meta-analysis are unlikely to be influenced by publication bias.

## Discussion

This systematic review provides a comprehensive synthesis of recent RCTs, employing meta-analysis to examine the impact of pulmonary rehabilitation, including exercise and breathing training, on postoperative recovery in lung cancer patients. The findings highlight several benefits, such as improved lung function, exercise capacity, and QoL.

### Lung function

Lung function is a critical prognostic factor for lung cancer outcomes [40]. FVC and FEV1 are the most commonly used measurements for assessing airway obstruction [41], with higher values associated with better survival outcomes [40, 42]. The present analysis demonstrated overall improvements in FVC and FEV1 following pulmonary rehabilitation, consistent with previous systematic reviews [13, 43]. Subgroup analysis further indicated that exercise training, particularly aerobic exercise, significantly improved FVC and showed a trend of increasing FEV1, while a combination of aerobic and resistance exercises showed benefits for FEV1. Breathing training, however, was underrepresented in the included studies, with only two focused on this intervention. Additionally, no more than three studies were available for each subgroup, limiting the robustness of the findings. Subgroup analysis based on intervention duration revealed that programs lasting at least 12 weeks were more effective in improving both FVC and FEV1, suggesting the importance of sustained interventions. Supervised interventions also demonstrated effectiveness in improving FVC and FEV1. Regarding exercise intensity, only one study on HIIT and three on moderate intensity were available; moderate intensity showed a small effect on FEV1. Given the limited number of studies and heterogeneity, these subgroup findings should be interpreted cautiously.

### Exercise capacity

Exercise capacity is often adversely affected in lung cancer patients due to tumor burden and treatment-related effects. Another study showed that compared to healthy controls, impairments in exercise capacity are among the most profound challenges for post-surgical lung cancer patients [44]. VO_2_peak, an indicator of cardiopulmonary fitness, declines by 19–28% within 14 days following lung resection surgery [45]. These limitations are clinically significant, as VO_2_peak is associated with survival in lung cancer patients [46, 47]. Our analysis confirmed improvements in exercise capacity indices, including 6 MWD and VO_2_peak among post-operative lung cancer patients, supporting the findings of previous reviews [11, 13]. Specifically, the MD of 6 MWD with 27.55 m exceeded the minimum clinically significant range of 22–42 m reported for people with lung cancer [48]. However, moderate heterogeneity was observed, likely due to variations in intervention methods and duration. Only three studies incorporating both exercise and breathing training were included in the subgroup analysis, resulting in insufficient data to draw robust comparisons with exercises and breath training and control groups.

Although this study did not identify significant impacts of breathing exercises on the 6 MWD, a finding consistent with prior research [49], their potential benefits in other domains warrant further investigation. Additional studies are also needed to confirm the effectiveness of combining breathing exercises with physical training in improving 6 MWD among postoperative lung cancer patients. Notably, subgroup analysis revealed that interventions lasting 12 weeks or longer were more effective in enhancing 6 MWD, emphasizing the importance of sustained rehabilitation programs. Unsupervised interventions also improved 6 MWD, but this subgroup included only three studies and had high heterogeneity, limiting robust comparison with supervised interventions. For exercise intensity, moderate intensity was effective for 6 MWD, but only one HIIT study was available. Therefore, these subgroup analyses should be viewed with caution.

### Pain and Dyspnoea

Pain and dyspnoea are common symptoms in lung cancer patients, significantly contributing to decreased postoperative QoL [50–52]. Our meta-analysis did not identify significant overall improvements in these symptoms following pulmonary rehabilitation. However, subgroup analyses revealed notable findings: interventions combining exercise and breathing training demonstrated potential for alleviating dyspnoea, consistent with a prior study [13]. Additionally, the duration of the intervention proved to be a critical factor, with programs lasting 12 weeks or longer demonstrating superior efficacy in reducing dyspnoea. These results highlight the potential of integrated exercise and breathing interventions as a promising approach for managing dyspnoea in postoperative lung cancer patients, particularly when implemented over an extended period. Only three studies reported exercise intensity, with two HIIT studies showing improvement in dyspnoea. Further research is needed to investigate optimal exercise intensity for symptom relief.

### QoL

Our analysis revealed an overall improvement in the physical domain but not the mental domain of QoL after pulmonary rehabilitation intervention, consistent with previous studies [11, 13]. However, subgroup analyses by different types of rehabilitation intervention yielded noteworthy findings. Our study suggests that a combination of exercise and breathing training could improve both physical and mental QoL. The subgroup combining exercise with breathing showed a significant effect on physical (MD = 8.50) and mental (MD = 7.9) domains, exceeding the minimal important difference (MID) for the SF-36 (i.e., 3 to 5 points) [53]. This finding is important because people with lung cancer often experience poorer physical and mental QoL after surgery. Compared to the pre-operative baseline, participants had worse functions that persisted even up to two years after surgery [45, 54, 55]. More importantly, QoL has been found to have the strongest association with survival, with a 14% decrease in the hazard of death per 10% increase in global QoL in lung cancer patients [56, 57]. Previous studies have reported that breathing exercises significantly improve patients’ QoL by reducing anxiety scores. Furthermore, alleviating symptoms of pain and dyspnoea and increasing lung function and exercise capacity could all contribute to the improvement of QoL. A previous systematic review discussed that a shorter rehabilitation period might be one of the reasons for negative findings in postoperative exercise training improving QoL in patients receiving pulmonary resection [16]. The findings of our study indicate that interventions lasting no less than 12 weeks show greater improvements in both physical and mental domains. Subgroup analysis also indicated greater improvements with HIIT. Future research should emphasize the long-term impact of pulmonary rehabilitation with breathing exercises on QoL to deepen the understanding of their overall benefits. Due to the small number of included studies and heterogeneity attributed to variations in study design, patient demographics, and training protocols, further research may be needed to address these issues.

### Limitation

Physical conditions in people with lung cancer are adversely affected by several factors, including the tumor itself, coexisting treatment for the condition, such as adjuvant therapies (e.g., chemotherapy or radiation), which may negatively impact patients’ symptoms and physical status. Surgical procedures beyond VATS and thoracotomy, including segmentectomy, lobectomy, or pneumonectomy, can also influence the extent of surgery and postoperative outcomes, potentially modifying the effect of pulmonary rehabilitation [58, 59]. However, our systematic review did not assess the impact of these diseases and treatment methods. Besides clinical factors, other participant characteristics may independently influence outcomes regardless of the intervention, such as baseline physical fitness; psychological factors like motivation, anxiety, or depression; social support and access to facilities; and demographic factors such as age and sex [60, 61]. Our review also did not evaluate the influence of these factors. Additionally, intervention-related factors, including patient adherence to the exercise program, with or without an education component, timing of the intervention relative to surgery, and variation in supervision, are important but were not addressed in this review due to limited information and should be considered in future studies. Due to the limited available data and lack of in-depth exploration, rigorous conclusions cannot be drawn about the effects of different exercise intensities or supervision on lung function, physical capacity, and QoL in post-resection lung cancer patients. Further research is necessary to unify the pulmonary rehabilitation structure and clarify these effects. Another limitation is that subgroup analyses must be interpreted cautiously because of data heterogeneity and small sample sizes. For example, high heterogeneity was observed in the 6MWD and VO2 peak outcomes. Additionally, we did not investigate the maintenance of improvements in these physical and psychological outcomes in this review. Future studies should explore strategies to sustain these gains.

### Relevance for clinical practice

More importantly, health professionals should be educated on the health benefits of pulmonary rehabilitation for physical function and QoL, particularly for patients with lung cancer who have undergone surgery. Given their central role in patient education and their frequent interaction with patients during clinical practice, health professionals, such as nurses, are in a good position to recommend pulmonary rehabilitation practices, such as exercise and breath training, for at-home implementation. Health professionals can also emphasize the importance of adherence to pulmonary rehabilitation plans and advocate for healthy lifestyle choices among patients.

## Conclusions

Overall, our findings of this review demonstrate the positive effects of pulmonary rehabilitation, including exercise and breathing training, on postoperative recovery in lung cancer patients. The findings underscore significant improvements in lung function, exercise capacity, and QoL across the included studies. Notably, interventions lasting 12 weeks or longer were associated with greater improvements in FVC, FEV1, 6 MWD, QoL, and dyspnoea. In light of these findings, pulmonary rehabilitation incorporating exercise and breathing training should be more widely adopted to support postoperative recovery in lung cancer patients. However, the small sample sizes and data heterogeneity warrant cautious interpretation.

There is a clinical need to educate health professionals about the benefits of pulmonary rehabilitation in enhancing exercise capacity, respiratory function, and QoL. By doing so, health professionals may provide evidence-based recommendations to patients, promoting pulmonary rehabilitation as a viable strategy to mitigate the adverse physical and mental health effects of their illness. This approach can empower patients to achieve a better QoL despite their condition.

## Supplementary Information

Below is the link to the electronic supplementary material.

Supplementary file1 (DOCX 33 KB)

Supplementary file2 (DOCX 24 KB)

Supplementary file3 (DOCX 1.80 MB)

## Data Availability

Data supporting the findings of this review are available in the review article and supplementary files.
