# Supplementary material for: Effects of pulmonary rehabilitation in people with lung cancer after lung resection: a systematic review and meta-analysis
Source: J Cancer Surviv. 2026 Jan 30;20(Suppl 2):2307–36. doi: 10.1007/s11764-025-01952-9 (PMC13744506; doi:10.1007/s11764-025-01952-9)
Supplement: Supplementary file 2 [file 11764_2025_1952_MOESM2_ESM.docx]

**Supplementary Material 2 search strategy**

| Pubmed  318 | Search: (((((((((((Lung Neoplasms[Title/Abstract]) OR (lung cancer[Title/Abstract])) OR (lung neoplasm*[Title/Abstract])) OR (lung tumor*[Title/Abstract])) OR (lung adenocarcinoma[Title/Abstract])) OR (Lung carcinoma[Title/Abstract])) OR (Pulmonary Neoplasm[Title/Abstract])) OR (carcinoma non-small-cell lung[Title/Abstract])) OR (carcinoma small-cell lung[Title/Abstract])) OR ("Lung Neoplasms"[Mesh])) AND (((((((((((((Rehabilitation[Title/Abstract]) OR (pulmonary rehabilitation[Title/Abstract])) OR (respiratory rehabilitation[Title/Abstract])) OR (lung rehabilitation[Title/Abstract])) OR (physical fitness training[Title/Abstract])) OR (physical rehabilitation[Title/Abstract])) OR (respiratory therapy[Title/Abstract])) OR (respiratory exercise[Title/Abstract])) OR (resistance exercise[Title/Abstract])) OR (Exercise[Title/Abstract])) OR (physical activity[Title/Abstract])) OR (aerobic exercise[Title/Abstract])) OR ("Rehabilitation"[Mesh] OR "Rehabilitation Nursing"[Mesh]))) AND (("Randomized Controlled Trial" [Publication Type] OR "Randomized Controlled Trials as Topic"[Mesh]) OR (((randomized controlled trial[Title/Abstract]) OR (randomized clinical trial[Title/Abstract])) OR (randomized controlled study[Title/Abstract]))) Sort by: Most Recent |
| --- | --- |
| web of sci  927 | **Article** or **Review Article** (Document Types) and **English** (Languages)  TS= (Lung Neoplasms) OR TS= (lung cancer) OR TS= (lung neoplasm*) OR TS= (lung tumor*) OR TS=(lung adenocarcinoma) OR TS= (Lung carcinoma) OR TS= (Pulmonary Neoplasm) OR TS= (carcinoma non-small-cell lung ) OR TS=(carcinoma small-cell lung)  AND  TS=(Rehabilitation) OR TS= (pulmonary rehabilitation) OR TS= (respiratory rehabilitation) OR TS=(lung rehabilitation ) OR TS=(physical fitness training ) OR TS=(physical rehabilitation ) OR TS=(respiratory therapy ) OR TS=(respiratory exercise) OR TS=(resistance exercise ) OR TS=(Exercise ) OR TS=(physical activity ) OR TS=(aerobic exercise)  AND  TS=(randomized controlled trial) OR TS=(randomized clinical trial) OR TS=(randomized controlled study) |
| CIHAHL  161 | S12 S4 AND S8 AND S11  S11 S9 OR S10  S10 PT randomized controlled trial OR TI randomized controlled trial OR TI randomized clinical trial OR TI randomized controlled study OR AB randomized controlled trial OR AB randomized clinical trial OR AB randomized controlled study  S9 (MH "Randomized Controlled Trials+")  S8 S5 OR S6 OR S7  S7 AB Rehabilitation OR AB pulmonary rehabilitation OR AB respiratory rehabilitation OR AB lung rehabilitation OR AB physical fitness training OR AB physical rehabilitation OR AB respiratory therapy OR AB respiratory exercise OR AB resistance exercise OR AB Exercise OR AB physical activity OR AB aerobic exercise  S6 (MH "Rehabilitation, Pulmonary+")  S5 TI Rehabilitation OR TI pulmonary rehabilitation OR TI respiratory rehabilitation OR TI lung rehabilitation OR TI physical fitness training OR TI physical rehabilitation OR TI respiratory therapy OR TI respiratory exercise OR TI resistance exercise OR TI Exercise OR TI physical activity OR TI aerobic exercise  S4 S1 OR S2 OR S3  S3 AB Lung Neoplasms OR AB lung cancer OR AB lung neoplasm* OR AB lung tumor* OR AB lung adenocarcinoma OR AB Lung carcinoma OR AB Pulmonary Neoplasm OR AB carcinoma non-small-cell lung OR AB carcinoma small-cell lung  S2 TI Lung Neoplasms OR TI lung cancer OR TI lung neoplasm* OR TI lung tumor* OR TI lung adenocarcinoma OR TI Lung carcinoma OR TI Pulmonary Neoplasm OR TI carcinoma non-small-cell lung OR TI carcinoma small-cell lung  S1 (MH "Lung Neoplasms+") |
| Embase  390 | 1 exp lung cancer/  2 exp lung tumor/  3 ("Lung Neoplasms" or "lung cancer" or "lung neoplasm* " or "lung tumor*" or "lung adenocarcinoma " or "Lung carcinoma" or "Pulmonary Neoplasm" or "carcinoma non-small-cell lung" or "carcinoma small-cell lung").ab,ti.  4 1 or 2 or 3  5 exp pulmonary rehabilitation/  6 ("Rehabilitation" or "pulmonary rehabilitation" or "respiratory rehabilitation" or "lung rehabilitation" or "physical fitness training" or "physical rehabilitation" or "respiratory therapy" or "respiratory exercise" or "resistance exercise" or "Exercise" or "physical activity" or "aerobic exercise").ab,ti.  7 5 or 6  8 exp randomized controlled trial/  9 ("randomized controlled trial" or "randomized clinical trial" or "randomized controlled study").ab,ti.  10 8 or 9  11 4 and 7 and 10 |
| Scopus  3434 | ( ( TITLE-ABS-KEY ( randomized AND controlled AND trial ) OR TITLE-ABS-KEY ( randomized AND clinical AND trial ) OR TITLE-ABS-KEY ( randomized AND controlled AND study ) ) ) AND ( ( TITLE-ABS-KEY ( rehabilitation ) OR TITLE-ABS-KEY ( pulmonary AND rehabilitation ) OR TITLE-ABS-KEY ( respiratory AND rehabilitation ) OR TITLE-ABS-KEY ( lung AND rehabilitation ) OR TITLE-ABS-KEY ( physical AND fitness AND training ) OR TITLE-ABS-KEY ( physical AND rehabilitation ) OR TITLE-ABS-KEY ( respiratory AND therapy ) OR TITLE-ABS-KEY ( respiratory AND exercise ) OR TITLE-ABS-KEY ( resistance AND exercise ) OR TITLE-ABS-KEY ( exercise ) OR TITLE-ABS-KEY ( physical AND activity ) OR TITLE-ABS-KEY ( aerobic AND exercise ) ) ) AND ( ( TITLE-ABS-KEY ( lung AND neoplasms ) OR TITLE-ABS-KEY ( lung AND cancer ) OR TITLE-ABS-KEY ( lung AND neoplasm* ) OR TITLE-ABS-KEY ( lung AND tumor* ) OR TITLE-ABS-KEY ( lung AND adenocarcinoma ) OR TITLE-ABS-KEY ( lung AND carcinoma ) OR TITLE-ABS-KEY ( pulmonary AND neoplasm ) OR TITLE-ABS-KEY ( carcinoma AND non-small-cell AND lung ) OR TITLE-ABS-KEY ( carcinoma AND small-cell AND lung ) ) ) AND ( LIMIT-TO ( DOCTYPE , "ar" ) OR LIMIT-TO ( DOCTYPE , "re" ) ) AND ( LIMIT-TO ( LANGUAGE , "English" ) OR LIMIT-TO ( LANGUAGE , "Chinese" ) ) |
