# Supplementary material for: Effects of pulmonary rehabilitation in people with lung cancer after lung resection: a systematic review and meta-analysis
Source: J Cancer Surviv. 2026 Jan 30;20(Suppl 2):2307–36. doi: 10.1007/s11764-025-01952-9 (PMC13744506; doi:10.1007/s11764-025-01952-9)
Supplement: Supplementary file 3 [file 11764_2025_1952_MOESM3_ESM.docx]

 **Supplementary Figure 1 Forest plot of changes in pain scores.**

 **Supplementary Figure 2 Forest plot of changes in Dyspnoea scores.**

**Supplementary Figure 3 Forest plot of comparing different interventions on FVC.**

**Supplementary Figure 4 Forest plot of comparing the different exercise types on FVC.**

**Supplementary Figure 5 Forest plot of comparing intervention duration on FVC.**

**Supplementary Figure 6 Forest plot of comparing intervention supervision on FVC.**

**Supplementary Figure 7 Forest plot of comparing different interventions on FEV1.**

**Supplementary Figure 8 Forest plot of comparing different exercise types on FEV1.**

**Supplementary Figure 9 Forest plot of comparing intervention duration on FEV1**

**Supplementary Figure 10 Forest plot of comparing intervention supervision on FEV1.**

**Supplementary Figure 11 Forest plot of comparing intervention intensity on FEV1.**

**Supplementary Figure 12 Forest plot of comparing different interventions on 6 MWD**

**Supplementary Figure 13 Forest plot of comparing intervention durations on 6 MWD**

**Supplementary Figure 14 Forest plot of comparing intervention supervision on 6 MWD.**

**Supplementary Figure 15 Forest plot of comparing intervention intensity on 6MWD.**

**Supplementary Figure 16 Forest plot of comparing different interventions on physical QoL**

**Supplementary Figure 17 Forest plot of comparing the different interventions on mental QoL**

**Supplementary Figure 18 Forest plot of comparing intervention durations on physical QoL**

**Supplementary Figure 19 Forest plot of comparing intervention duration on mental QoL**

**Supplementary Figure 20 Forest plot of comparing intervention intensity on physical QoL.**

**Supplementary Figure 21 Forest plot of comparing intervention intensity on mental QoL.**

**Supplementary Figure 22 Forest plot of comparing different interventions on dyspnea**

**Supplementary Figure 23 Forest plot of comparing interventions duration on dyspnoea**

**Supplementary Figure 24 Forest plot of comparing intervention supervision on dysponea.**

**Supplementary Figure 25 Forest plot of comparing intervention intensity on dysponea.**
